# Supplementary material for: Causal Relationship between Aging and Anorexia Nervosa: A White-Matter-Microstructure-Mediated Mendelian Randomization Analysis
Source: Biomedicines. 2024 Aug 16;12(8):1874. doi: 10.3390/biomedicines12081874 (PMC11351342; doi:10.3390/biomedicines12081874)
Supplement: Supplementary file 1 [file biomedicines-12-01874-s001.zip › Supplementary Figure/Figure S4/Singe SNP plot of MR analysis for TL to ubm-b-2050.pdf]

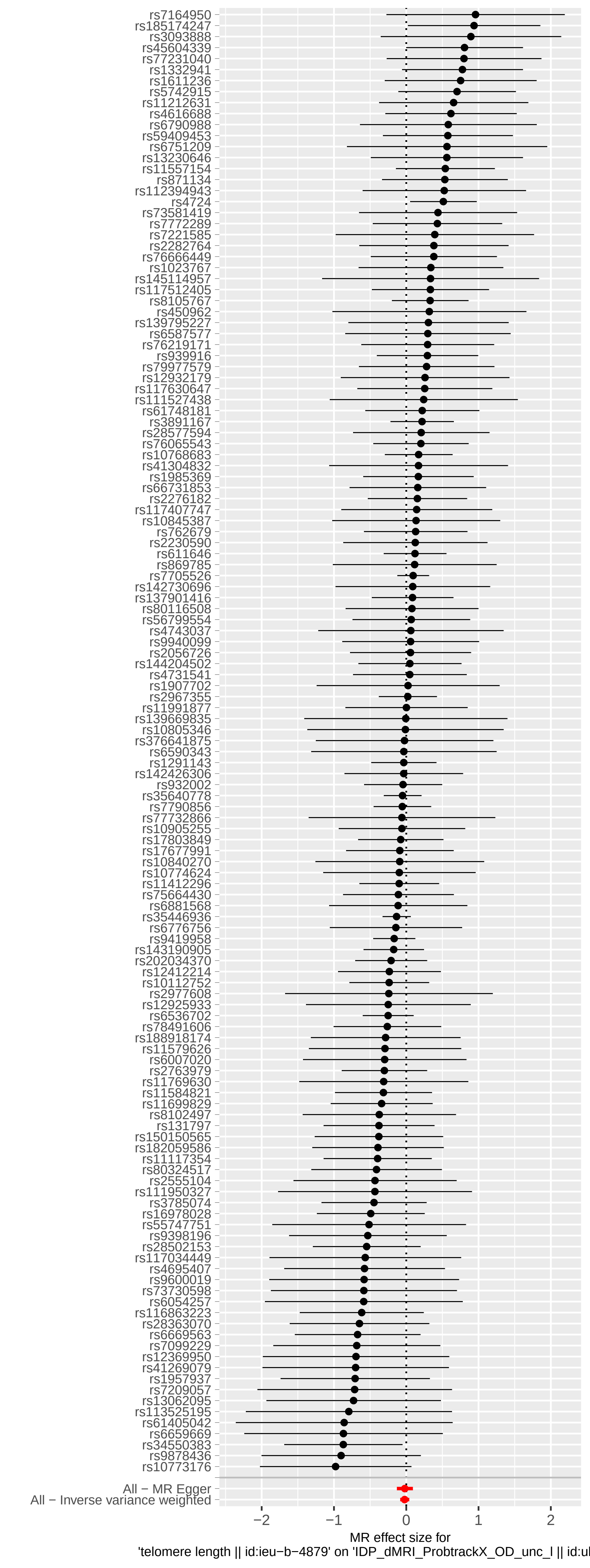

rs7164950
rs185174247
rs3093888
rs45604339
rs77231040
rs1332941
rs1611236
rs5742915
rs11212631
rs4616688
rs6790988
rs59409453
rs6751209
rs13230646
rs11557154
rs871134
rs112394943
rs4724
rs73581419
rs7772289
rs7221585
rs2282764
rs76666449
rs1023767
rs145114957
rs117512405
rs8105767
rs450962
rs139795227
rs6587577
rs76219171
rs939916
rs79977579
rs12932179
rs117630647
rs111527438
rs61748181
rs3891167
rs28577594
rs76065543
rs10768683
rs41304832
rs1985369
rs66731853
rs2276182
rs117407747
rs10845387
rs762679
rs2230590
rs611646
rs869785
rs7705526
rs142730696
rs137901416
rs80116508
rs56799554
rs4743037
rs9940099
rs2056726
rs144204502
rs4731541
rs1907702
rs2967355
rs11991877
rs139669835
rs10805346
rs376641875
rs6590343
rs1291143
rs142426306
rs932002
rs35640778
rs7790856
rs77732866
rs10905255
rs17803849
rs17677991
rs10840270
rs10774624
rs11412296
rs75664430
rs6881568
rs35446936
rs6776756
rs9419958
rs143190905
rs202034370
rs12412214
rs10112752
rs2977608
rs12925933
rs6536702
rs78491606
rs188918174
rs11579626
rs6007020
rs2763979
rs11769630
rs11584821
rs11699829
rs8102497
rs131797
rs150150565
rs182059586
rs11117354
rs80324517
rs2555104
rs111950327
rs3785074
rs16978028
rs55747751
rs9398196
rs28502153
rs117034449
rs4695407
rs9600019
rs73730598
rs6054257
rs116863223
rs28363070
rs6669563
rs7099229
rs12369950
rs41269079
rs1957937
rs7209057
rs13062095
rs113525195
rs61405042
rs6659669
rs34550383
rs9878436
rs10773176
All − MR Egger
All − Inverse variance weighted
−2
−1
0
1
2
MR effect size for
'telomere length || id:ieu−b−4879' on 'IDP_dMRI_ProbtrackX_OD_unc_l || id:ul
